# Complete Pathological Response to Neoadjuvant Treatment in Pancreatic Ductal Adenocarcinoma—Can We Achieve a Long-Term Survival? A Narrative Review

**DOI:** 10.3390/life15121833

**Published:** 2025-11-28

**Authors:** Magdalena Gajda, Ewa Grudzińska, Łukasz Liszka, Joanna Pilch-Kowalczyk, Sławomir Mrowiec

**Affiliations:** 1 Department of Gastrointestinal Surgery, Medical University of Silesia, 40-752 Katowice, Poland; ewa.grudzinska@sum.edu.pl (E.G.); smrowiec@sum.edu.pl (S.M.); 2 Department of Pathomorphology and Molecular Diagnostics, Medical University of Silesia, 40-752 Katowice, Poland; katpat2@sum.edu.pl; 3 Department of Radiodiagnostics and Interventional Radiology, Medical University of Silesia, 40-752 Katowice, Poland; sekretariatrtg@uck.katowice.pl

**Keywords:** pancreatic adenocarcinoma, long-term survival, neoadjuvant therapy, complete pathological response, surgical resection

## Abstract

Pancreatic ductal adenocarcinoma (PDAC) is one of the leading causes of cancer-related mortality in Europe, with a 5-year survival rate of approximately 10%. Surgical intervention is the only curative method of treatment in PDAC. However, especially in the case of patients with borderline or locally advanced cancer, neoadjuvant treatment is often administered in an attempt to downstage the tumor. Uncommonly, after neoadjuvant treatment, no viable tumor in the specimen after surgical resection is found- this is defined as a complete pathological response (pCR). Our paper presents a narrative review of this rare phenomenon and its possible association with patient’s survival. Conclusions: Achieving pCR may be associated with a significant improvement in the prognosis of patients with PDAC. However, it remains unknown why pCR is achievable in only a few patients. Further studies on large groups of patients are needed to identify the factors that increase the chance of pCR.

## 1. Introduction

Pancreatic ductal adenocarcinoma (PDAC) has a very high mortality and a poor 5-year survival rate of approximately 10% [1]. Globally, the incidence of pancreatic cancer is predicted to increase to 18.6 per 100,000 in 2050, with an average annual increase of 1.1%, which makes it an important issue for public health [2]. Symptoms of PDAC in its early stages are scant, so it is usually advanced when detected [3]. PDAC includes potentially resectable cases (PR-PDAC), borderline resectable cases (BR-PDAC), and locally advanced disease (LA-PDAC, unresectable)—this classification is based on the degree of arterial and venous tumor involvement [4].

Surgical intervention is the only potentially curative method of treatment in pancreatic cancer. However, the role of preoperative treatment—neoadjuvant treatment (NAT) is becoming increasingly important in PR-PDAC [5,6], BR-PDAC [7,8], and LA-PDAC cases [9]. NAT and involves chemotherapy, radiotherapy, or chemoradiation [9].

In clinical practice, for now, NAT is broadly accepted only for BR-PDAC, while upfront surgery is still the recommendation for PR-PDAC except in cases with high-risk features, including equivocal or indeterminate imaging findings, markedly elevated carbohydrate antigen 19-9 (CA19-9), large tumors and lymph nodes, excessive weight loss, and intense pain [10]. NAT is intended to increase the rate of R0 resections [8], reduce the tumor to a resectable state [9], treat micrometastases that may be undetectable on computed tomography (CT) [9,11], and improve overall survival [12]. According to the current Protocol for the Examination of Specimens From Patients with Carcinoma of the Pancreas [13], the tumor response to NAT should be included in the pathological report. When no viable cancer cells are found in the specimen, a complete pathological response (pCR) is reported.

The prognostic value of pathological response after NAT was clarified in the breast, rectal, and esophageal carcinoma, where pCR was associated with a lower recurrence rate and prolonged survival [14,15,16]. The role of pCR in improving survival in PDAC is currently unclear.

In this article, we review the literature regarding the possibility of achieving pCR, and its association with the patient’s prognosis, with particular emphasis on long-term survival in PDAC. We also indicate potential directions for future research on achieving pCR.

This is a narrative review; therefore, PRISMA methodology was not used, which may have resulted in selection bias. However, we made every effort to conduct a comprehensive literature search using PubMed and Google Scholar (using keywords such as “complete pathological response in pancreatic cancer”, “neoadjuvant therapy in pancreatic cancer,” “long-term survival in pancreatic cancer”, etc.) and included the most relevant publications (meta-analyses, retrospective cohort studies, and case reports) regarding human research, published in English, over a 20-year period (2005–2025).

## 2. Definition and Incidence of pCR

Tumor response to NAT should be reported in every pathological report. Multiple scoring systems have been created [17,18]. Nowadays, the College of American Pathologists (CAP) recommends a modified Ryan scheme (Table 1), as it has been shown to be a prognostic factor for overall survival (*p* = 0.043) [19].

The occurrence of pCR after pancreatic resection is rarely reported in the literature. In PDAC patients receiving NAT, the percentage reaches several percent. In a meta-analysis, it was 4–7%; in prospective studies, 0–9.09%; and in prospective databases, 1.63–11.11% [20,21].

In the retrospective cohort studies we analyzed, the incidence of pCR was 0.8% to 14.5% (Table 2)—in the vast majority of reported studies, achieving pCR was associated with a longer survival.

**Figure 1:**
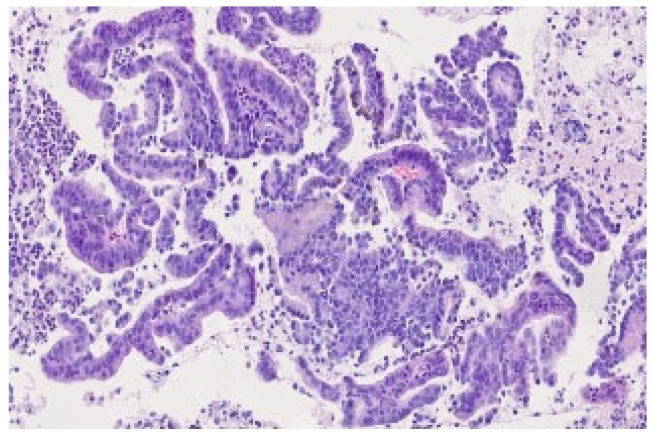
PDAC detected in fine needle biopsy during endoscopic ultrasonography (EUS) in the 65-year-old patient with a tumor of the pancreatic head (BR-PDAC). We can see lobules of ductal adenocarcinoma. Hemotoxylin and eosin stain, 20× enlargement. The photo comes from the own material of the Department of Pathomorphology and Molecular Diagnostics, Medical University of Silesia, Katowice, Poland.

**Figure 2:**
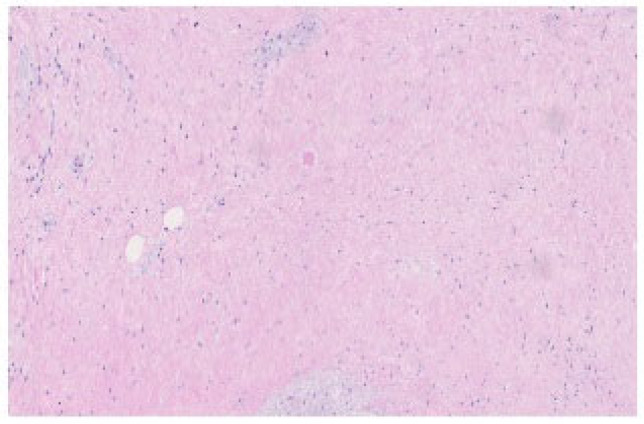
The postoperative specimen after 6 cycles of neoadjuvant chemotherapy according to the FOLFIRINOX regimen in the 65-year-old patients with a BR-PDAC– no visible cancer cells in the microscopic view—pCR. Hemotoxylin and eosin stain, 20× enlargement. The photo comes from the own material of the Department of Pathomorphology and Molecular Diagnostics, Medical University of Silesia, Katowice, Poland.

Our institution noted only one case of pCR per 258 cases after NAT in the last 8 years (0.38%)—a 65-year-old patient with BR-PDAC diagnosed during endoscopic ultrasonography (Figure 1). After 6 cycles of neoadjuvant chemotherapy according to the FOLFIRINOX regimen, we performed a pancreaticoduodenectomy (PD), and achieved pCR (Figure 2). Two years after surgery, the patient is alive, and without any signs of disease recurrence.

## 3. Biological Mechanisms Leading to pCR

The biological mechanisms leading to pCR in PDAC are not fully understood. They involve a combination of tumor genetic features—such as defects in DNA repair pathways that increase sensitivity to chemotherapy—and the host immune response, including increased immune cell infiltration and alterations in immune pathways within the tumor microenvironment [42,43]. Some studies indicate that neoadjuvant therapy reduces tumor-infiltrating cells associated with immunosuppression and poor survival by lowering the levels of FoxP3+ regulatory T cells and myeloid cells compared with tumors primarily resected [44]. Additionally, the important role of the local immune response associated with the presence of high-quality neoantigens in long-term survivors [45] highlights a potentially promising direction for further research on the roles and types of immune cells involved in achieving pCR.

Other biological mechanisms that may play a role in achieving pCR include inflammatory markers, such as lymphocyte count, monocyte count, neutrophil-to-lymphocyte ratio (NLR), platelet-to-lymphocyte ratio (PLR), and systemic inflammatory response index (SIRI). Their significance has been studied in breast cancer [46], where elevated lymphocyte counts (*p* = 0.004) were connected to a higher rate of pCR. A lower pCR rate was associated with increased monocyte count (*p* = 0.006), NLR (*p* = 0.005), PLR (*p* = 0.005), SIRI (*p* = 0.037), systemic immune-inflammation index (*p* = 0.029), and preoperative SIRI (*p* = 0.010). In PDAC, the impact of the following parameters on prognosis was examined: NLR—a low NLR was associated with a longer OS [47]; PLR—low PLR was associated with a longer OS and PFS [48]; lymphocyte-to-monocyte ratio (LMR)—a low LMR was associated with poor OS in meta-analyses [49,50], and SIRI—in a meta-analysis [51] of seven studies including 1160 patients, a higher SIRI was associated with worse OS and PFS. Unfortunately, there are no studies examining the impact of the above inflammatory markers on achieving pCR in PDAC; however, considering their potential significance, this is certainly a direction worth investigating in future research.

Circulating tumor DNA (ctDNA) may also have a potentially useful role in pCR achievement. These are small parts of DNA released into the bloodstream from the neoplasm. They are available for non-invasive testing and are regarded as a significant predictor of treatment response in PDAC, also correlating with tumor burden and stage [52]. Additionally, ctDNA may provide information on the molecular profile and biological characteristics of PDAC [53]. Monitoring ctDNA during and after therapy provides a good picture of treatment response. Reductions in ctDNA levels occurring during therapy might predict a higher chance of achieving pCR.

## 4. The Influence of pCR on OS and PFS

The consequences of pCR for patients are ambiguous, but many publications report remarkable results in terms of prolonged OS and PFS in these patients (Table 2). The study by Bao [24] confirmed that greater pathological response is associated with longer OS (OR 0.50 95% CI 0.29–0.88, *p* = 0.01). In this study, nCR was observed in 52 (26.0%) and pCR in 15 (7.3%) patients, respectively, and the 1-, 3- and 5-year OS were 92.7, 67.2 and 41.7%, and 71.0, 37.4 and 20.8% in patients with or without major pathological response, respectively (log-rank test *p* < 0.001). In the study by Cloyd et al. [25], the longer OS median was associated with greater pathological response (73.4 months in patients who achieved vs. 32.2 months than in those who did not, *p* < 0.001). According to Zhao et al. [27], patients with pCR had better OS than those who had tumor cells in the specimen after treatment (*p* < 0.001).

In the Sell et al. publication [29], the median OS was 43 months in patients with pCR, 24 months in those with nCR, and 23 months in those with PRsp (*p* < 0.0001). Only pCR was associated with improved OS in adjusted Cox regression. In turn, He et al. [30] reported that the median PFS was 26 months in patients with pCR, which was longer than in patients with nCR (12 months, *p* = 0.019) and PRsp (12 months, *p* < 0.001). The median OS for nCR (27 months, *p* = 0.003) or PRsp (26 months, *p* = 0.001) was lower than for pCR (over 60 months). In multivariate analysis, pCR was an independent prognostic factor for PFS (HR = 0.45; 0.22–0.93, *p* = 0.030) and OS (HR = 0.41; 0.17–0.97, *p* = 0.044).

In the study by Blair et al. [31] pCR after NAT in patients with LA-PDAC was associated with extremely long survival, although approximately half of the patients had a relapse. In this analysis, the median PFS in pCR patients was 29 months, while OS was 76 months. In the study by Barrak et al. [34], pCR was connected to a significant increase in OS, with a 64.9-month median survival. Lack of pCR meant a median survival of only 21.6 months (*p* < 0.0001). According to this study, pCR is associated with improved OS compared to patients with residual disease. Mellon et al. [35] compared PatR levels (TRG 1-3, median OS 33.9 months, median PFS 13.0 months), with six (7%) patients with TRG 0. The latter group had zero mortality (*p* = 0.05), with only one progression (*p* = 0.03).

In his work, Ahn [19] demonstrated that the CAP staging system is a significant prognostic factor for OS (*p* = 0.043). The four-stage CAP tumor pathologic regression grading system accurately predicts clinical outcomes in LAPC patients undergoing radical surgery after NAT. However, in Truta’s report [36], only three independent factors for prolonged survival were described, including longer chemotherapy (≥6 cycles), CA19-9 decline after chemotherapy, and major pathological response. Patients who achieved all three parameters showed improved survival outcomes, and reduced survival was noted with each failed parameter.

In Tamburrino’s work [38], a total of 403 patients who underwent NAT and surgery for PDAC were examined. After a 42-month follow-up (95% CI 38–45), 3-year disease-specific survival was 87% in patients with pCR compared with 43% in those without pCR (*p* = 0.014). The relapse rate was 40% (*n* = 6/15) in the pCR group compared with 69.8% (*n* = 271/388) in the non-pCR group. pCR group is associated with longer PFS, with higher 1- and 3-year rates compared with the non-pCR group (80% vs. 60% and 48% vs. 24%, respectively). pCR was an independent protective factor for PFS (*p* = 0.035). According to the authors, pCR in PDAC does not equate to a cure, but is associated with longer OS.

In turn, other reports suggest that pCR to NAT in BRPC does not indicate a statistically significant improvement in survival. Peng showed [33] the median OS of 50.0 months (pCR or nCR), 31.7 months (PRsp), and 23.2 months (Nsp) (*p* = 0.563). The study by Hashemi-Sadraei et al. [37] concluded that the pCR result does not indicate a complete cure, and most patients experience local or systemic recurrence. Also in the Bolton et al. study, pathological response (CAP grade 0 or 1) was not associated with survival (*p* = 0.13) [54].

Most studies highlight improved survival when pCR is achieved, although individual reports do not share these observations. The impact of pCR on OS and PFS should be investigated in further multicenter studies.

## 5. The Influence of the Type of NAT on the Possibility of Achieving pCR

### 5.1. Role of Chemotherapy and Chemoradiotherapy

In cases of BD-PDAC and LA-PDAC, oncological guidelines [10,55] recommend the inclusion of NAT because upfront surgery has a high risk of positive surgical margins [56], which may significantly worsen the treatment results [56,57]. The introduction of NAT had positive effects on patients with BD-PDAC and LA-PDAC.

Gillen’s review of 111 studies [58] led the authors to a conclusion that one-third of patients with initially unresectable LA-PDAC may achieve resectability following NAT (including radiotherapy and/or chemotherapy).

Currently, many scientific studies investigate factors that increase the probability of achieving pCR. One of the areas that this research focuses on is the type of NAT used.

Several reports highlight the value of total neoadjuvant therapy (TNT). This term refers to the administration of both chemotherapy (ChT) and chemoradiation (CRT) (radiation initiated at least 28 days after chemotherapy start) before definitive resection. In the study by Barrak et al. [34], a multivariate analysis showed that the probability of pCR was higher in the case of TNT than CRT (OR 1.67, CI 1.13–2.46, *p* = 0.0103) or ChT (OR 2, 61, CI 1.83–3.71, *p* < 0.0001), which means that TNT may be a beneficial option for patients requiring NAT, as it may be associated with an increased possibility of achieving a pCR, thus potentially improving OS (median survival of 64.9 months for pCR vs. 21.6 months for the non-pCR group, *p* < 0.0001).

The value of TNT in achieving pCR was also highlighted in the work of Villano et al. [59], where it was shown that pCR occurred more often in the TNT group (10.1%) compared to the ChT and/or CRT (3.6%) or upfront surgery (0.6%) group (*p* = 0.001), which was associated with prolonged survival (median OS = 100.2 months).

Other authors emphasize the role of CRT and CR in NAT. In He et al. [30], multivariate logistic regression showed that CRT was associated with an increased occurrence of pCR or nCR. In this study, however, neither the chemotherapy type nor its length was associated with pathological response. In turn, Rajagopalan et al. [60] reported that after stereotactic body radiation therapy (SBRT) 25% (*n* = 3/12) of patients achieved a pCR, and an additional 16.7% (*n* = 2/12) showed <10% viable cancer cells. It must be noted that this analysis included a limited group of patients (*n* = 12) and requires verification in further research.

The positive effects of using FOLFIRINOX scheme as NAT are reported in many studies [61,62,63], which emphasize a favorable median OS and an increase in the resection rate after this type of NAT. The effect of FOLFIRINOX on achieving pCR is also the subject of many reports. Available publications are most often case reports on the achievement of a pCR result after using FOLFIRINOX as NAT in the case of BD-PDAC [64] or LA-PDAC [65]. However, the study by Jeon et al. [40] analyzed the clinical course in terms of pathological response in patients who received FOLIFIRNOX in the treatment of advanced PDAC. Of the 64 patients, 8 (12.5%) achieved pCR, and 8 (12.5%) had nCR. In the fPatRsp group, median OS and PFS were longer compared to the ufPatRsp group: over 60 months vs. 38 months, *p* < 0.001; over 42 months vs. 10 months, *p* < 0.001. In multivariate analyses, fPatRsp and adjuvant therapy were independent prognostic factors for OS (HR: 0.12; 95% CI: 0.02–0.96, *p* = 0.05; HR: 0.26; 95% CI: 0.09–0.74, *p* = 0.01) and PFS (HR: 0.31; 95% CI: 0.12–0.86, *p* = 0.02; 95% CI: 0.13–0.72, *p* = 0.01). According to the authors, type of pathological response may be associated with survival after pancreatectomy following the neoadjuvant FOLFIRINOX for PDAC. The authors also emphasize the role of adjuvant treatment.

In 2024, the results of an international cohort study assessing the rate of pCR after preoperative chemo(radio)therapy for pancreatic cancer were published [66]. It included patients who underwent surgery after ≥2 cycles of chemotherapy (with or without radiotherapy) at 19 centers in 8 countries, between 2010 and 2018. The median follow-up was 19 months. The rate of pCR, its association with postoperative OS, and factors associated with achieving pCR were assessed. A total of 1758 patients (mean age 64 years; men 50%) were included. The rate of pCR was 4.8% (*n* = 85), and pCR was associated with a longer OS (HR 0.46; 95% CI: 0.26–0.83). Overall survival at 1, 3, and 5 years was 95%, 82%, and 63%, respectively, in patients with pCR, compared with 80%, 46%, and 30%, respectively, in patients who did not achieve pCR (*p* < 0.001 The achievement of pCR was associated with preoperative multiagent chemotherapy other than (m)FOLFIRINOX (OR 0.48; 95% CI: 0.26–0.87), preoperative conventional radiotherapy (OR 2.03; 95% CI: 1.00–4.10), preoperative SBRT (OR 8.91; 95% CI: 4.17–19.05), radiological response to therapy (OR 13.00; 95% CI: 7.02–24.08), and normalization of Ca 19–9 antigen levels following preoperative therapy (OR 3.76; 95% CI: 1.79–7.89). The study authors report that pCR occurred in 4.8% of patients who underwent resection of PDAC after preoperative chemo(radio)therapy. It must be remembered that pCR does not imply a complete cure; however, it does improve the OS, which at 5 years is >2-fold greater (63% vs. 30%) than in those without pCR.

Analyzing the available literature, the impact of the type of NAT on the probability of achieving pCR is ambiguous and requires clarification in subsequent studies. At this point, there are no clear recommendations concerning the choice of the optimal NAT to achieve a pCR.

### 5.2. Role of Immunotherapy

In some cancers (melanoma [67], triple-negative breast cancer [68], renal cell carcinoma [69], non-small cell lung cancer [70]), neoadjuvant immunotherapy enables active stimulation of the immune system, which induces a stronger T-cell response, targeting the entire tumor. The immunotherapy is the form of immune checkpoint inhibitors (ICIs) that target antibodies against programmed cell death protein 1/programmed cell death ligand 1 (PD-1/PD-L1). After resection, tumor-specific T cells may remain active, further eliminating residual disease and micrometastases, which in turn may lead to improved cure rates [71].

Given the important role of immunotherapy in preoperative treatment, new immunological pathological response criteria have been proposed for some cancers, such as melanoma, and specifically for patients treated with neoadjuvant immunotherapy. This pathological scoring system is designed to assess outcomes following neoadjuvant PD-1/PD-L1 inhibitor administration. Tumor response is assessed in these criteria based on the percentage of immune-related residual tumor volume (%irRTV), established by dividing the residual tumor area by the total tumor bed area (the sum of the residual tumor area, necrosis area, and regression area) [67].

In PDAC, the assessment of pathological response (Table 1) is not dependent on immune-mediated tumor regression, as immunological therapy in pancreatic cancer is not widely used. However, the importance of recent research findings should be emphasized.

In the study by Abaydulla E et al. [39], patients with NAT with immunotherapy—PD-1 blockade plus CRT (combined group) with very high pCR rate (23.1%), was associated with a higher 2-year OS rate (75.2%), and longer median OS (30.5 months) compared to non-combined group (PD-1 + CT) where pCR rate was 0%, and 2-year OS rate was 42.6%, while median OS was 23.3 months. However, in this study 2-year OS rate and median OS were not significant between groups (*p* = 0.253 and *p* = 0.473). One of the reasons may be the small sample size and retrospective nature of this study. pCR was not examined as a single factor influencing OS in this study, although all pCR cases occurred in the extended survival group (only in the combined group). The very high pCR rate achieved in this study (the highest to our knowledge) may be due to increased PD-L1 expression induced by CRT, making this treatment regimen more effective [72]. The use of chemotherapy and radiotherapy, in addition to their cytotoxic function, also has an immunomodulatory function—such conclusions appear in several studies concerning the combination of immunotherapy with standard chemotherapy/radiotherapy in the therapy of solid tumors [73]. These important observations emphasize that achieving pCR requires complex preoperative management, combining immunotherapy and CRT regimens.

This is certainly an issue that needs clarification in future research on PDAC. When using immunotherapy, the currently used Tumor Regression Grading System may prove insufficient and may need to be modified, with the need to determine the %irRTV, similar to those used in melanoma.

## 6. The Influence of Other Factors on Achieving pCR

In addition to NAT, other factors that may influence the probability of achieving pCR have been investigated. In the study by Yeung et al. [74], in which the authors analyzed 10 patients with pCR, a significant decrease in CA 19-9 after NAT was noted: the mean serum CA 19-9 level before NAT was 148.0 ± 146.3 IU/L, and after NAT it was 18.0 ± 18.7 IU/L (*p* = 0.01). In the work by Perri et al. [32] in multivariate logistic regression, low CA 19-9 concentrations after the treatment, partial RECIST (Response Evaluation Criteria In Solid Tumors) response, and tumor volume reduction were found to be independently associated with better pathological response (*p* < 0.01). Also in the study by Mellon et al. [35], the decrease in CA 19-9 after NAT was associated with the improvement in TRG (*p* = 0.02). The study by Bao [24] also showed that normalization of CA19-9 after treatment (OR 4.20, 95% CI 1.14–10.35, *p* = 0.02) was associated with a fPatRsp. Another predictive factor in this study was radiological evidence of residual tumor size < 25 mm (OR 2.71, 95% CI 1.27–5.79, *p* = 0.01) after the treatment. The issue of tumor size after NAT was also examined by Servin-Rojas [22], where regression of tumor size after NAT was associated with an increased likelihood of a fPatRsp.

There are also single reports of the influence of race on the possibility of achieving pCR. In the Ogobuiro study [75], black race was independently associated with less frequent favorable pathological outcomes (OR 0.26, 95% CI 0.10–0.69). Black patients undergoing pancreatectomy appeared to be less likely to have a fPatRsp after NAT, although there was no significant difference in PFS or OS between the cohorts depending on race.

Shoucair et al. [76] analyzed biopsy samples and showed that lower matrix metalloproteinase 7 (MMP-7) expression was associated with a fPatRsp. The positive predictive value and negative predictive value of MMP-7 protein expression for ufPatRsp were 88.2% and 73.9%, respectively.

Mutations in germline BRCA1 and/or BRCA2 genes (gBRCAm) increase the risk of PDAC. They occur in approximately 10% of PDAC patients and are associated with increased DNA repair deficiency [77]. In the case of gBRCAm, studies support the efficacy of platinum treatment. Platinum, by cross-linking DNA strands, is destructive to DNA, leading to replication arrest and programmed death of cancer cells [78]. As reported by Golan et al. [79], patients with gBRCAm and BR-PDAC have an increased chance of pCR and longer OS after neoadjuvant FOLFIRINOX. The results confirmed the positive effect of treating gBRCAm patients with platinum chemotherapy early in the disease course. Therefore, according to the author, the neoadjuvant FOLFIRINOX should be considered in BRCA carriers and patients with BR-PDAC. The pCR rate was 44.4% in gBRCAm patients and 10% in BRCA non-carrier patients (*p* = 0.009). These conclusions were confirmed by Pinelli [80] in his case report, in which he described a PDAC patient with a BRCA2 mutation in the germline. The patient received first- and second-line chemotherapy and, after a year, underwent exploratory laparoscopy and total pancreatectomy with splenectomy. In the histopathological result, viable cancer cells were absent. After six months of follow-up, there was no recurrence of the disease.

**Figure 3:**
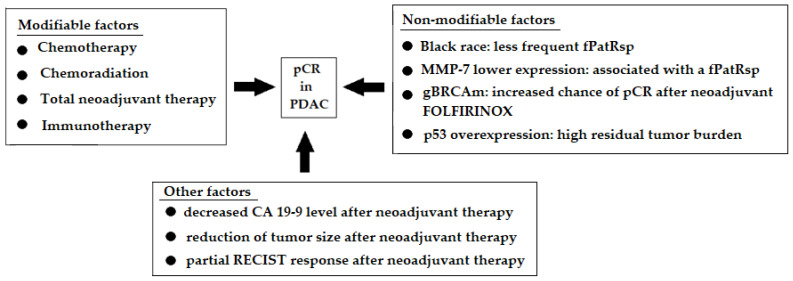
Summary of factors that may be associated with achieving pCR [22,23,24,30,32,34,35,39,40,58,59,60,66,74,75,76,79,80].

A transcription factor with tumor suppressor properties—p53 protein—may also influence the occurrence of pCR. In the study by White et al. [23], p53 overexpression was more common in patients with high residual tumor burden (Figure 3).

## 7. Achieving pCR Does Not Equate to a Cure

It should be clearly emphasized that achieving pCR does not mean complete recovery [37,38]. Although pCR may be associated with a favorable response to neoadjuvant therapy, it should not be interpreted as sufficient evidence of complete eradication of tumor cells. In accordance with current practice, it is a standard policy to administer the adjuvant treatment after NAT and surgical treatment in PDAC, regardless of the histopathological results of the removed tumor. Patients with PDAC, even after pCR is confirmed in the pathological specimen, continue oncological treatment. According to NCCN guidelines, patients should undergo adjuvant ChT or CRT [10]. PDAC is associated with a high risk of microscopic dissemination, and postoperative treatment aims to eliminate any remaining cells that may reside in the body despite apparently complete tumor removal. The CTCs may play a significant role in this phenomenon. The hypothesis is that the primary tumor cells infiltrate the nearest blood vessels, spread via the circulation, settle in distant sites, and thus cause secondary metastases [81]. Studies have shown that the CTC phenomenon correlates with the presence of occult metastases, early relapse, and overall survival in patients with PDAC [82,83,84]. It is possible that CTC-based testing will be used in the near future to monitor response to NAT or even predict which patients are likely to develop pCR, thus allowing for more selective oncological treatment choices [85].

## 8. Confounding Factors

The cited studies may be subject to confounding factors that could affect the conclusions. Tumor biology, including grading, may have a significant impact on achieving pCR. In breast cancer studies, patients with high-grade tumors had a higher chance of achieving a pCR [86]. This type of association in PDAC has not been investigated; this may be an interesting topic for future research. Selection bias may also play a significant role in the results, NAT assignment is a non-random assignment [10], potentially favoring healthier patients for NAT.

These confounding factors should be addressed in future studies examining the role of pCR in PDAC. The influence of confounding factors should be emphasized in future studies examining the role of pCR in PDAC.

## 9. Conclusions

Due to complicated biology of the PDAC, no single factor determines long-term survival, and recurrence may occur despite favorable prognostic factors. Although pCR does not mean a complete cure, when it is achieved, a significant improvement in the prognosis of PDAC patients is observed—several reports indicate an association between pCR and prolonged PFS and OS. Unfortunately, the factors that enable pCR to be obtained are unknown. Achieving pCR most likely requires a complex preoperative approach, combining chemoradiation and chemotherapy regimens. Modern immune-based treatments and techniques for detecting circulating tumor DNA may be the future of successful assessment of the NAT response, and perhaps even to predict which patients are likely to develop pCR, enabling a more selective and personalized oncological approach. Subsequent multicenter studies should focus on identifying the factors that increase the chance of pCR. Further work with this unique group of patients may contribute to better personalized therapeutic approaches for PDAC patients.

## Figures and Tables

**Table 1:** CAP Tumor Regression Grading System for Pancreatic Ductal Adenocarcinoma after Neoadjuvant Chemotherapy (TRG). In many studies, the results of TRG 0 and 1 are reported together and referred to as “favorable pathological response” (fPatRsp), and TRG 2 and 3 as “unfavorable pathological response” (ufPatRsp).

Tumor Regression Score	Description	Type of Pathological Response (PatRsp)	
0	No viable cancer cells	Complete response (pCR)	Favorable pathological response (fPatRsp)
1	Single cells od rare small groups of cancer cells	Near complete response (nCR)	
2	Residual cancer with obvious tumor regression, however, more than single cells or a few small groups of cancer cells	Partial response (PRsp)	Unfavorable pathological response (ufPatRsp)
3	Extensive residual cancer with no evident tumor regression	Poor or no response (Nsp)	

**Table 2:** pCR incidence and association with OS (overall survival) and/or PFS (progression-free survival) according to the available retrospective cohort studies. ^1^—in this study authors distinguished pCR group, but ultimately studied the pathologic major response group (pMR; <5% of residual viable cancer cells); ^2^—in the entire studied cohort (*n* = 47) the pCR rate was 12.76% (*n* = 6), but in the individual studied groups: 23.1% (*n* = 6) in combined group (26 patients who received neoadjuvant PD-1 blockade plus chemoradiotherapy) and 0.0% (*n* = 0) in non-combined group (21 patients who received PD-1 blockade plus chemotherapy); NR—not reported (the pCR group was not studied separately).

Author	Year	Frequency of pCR (%)	Number of pCR Cases (*n*)	Association with Prolonging Survival (OS and/or PFS)
Ahn S et al. [19]	2022	2.60%	1	yes
Servin-Rojas M et al. [22]	2024	4.00%	8	yes
White RR et al. [23]	2005	6.00%	4	yes
Bao QR et al. [24]	2023	7.30%	15	yes
Cloyd JM et al. [25]	2017	3.90%	23	yes
Xia BT et al. [26]	2017	2.63%	1	no
Zhao Q et al. [27]	2012	2.50%	11	yes
Gleeson EM et al. [28]	2021	7.30%	3	NR
Sell NM et al. [29]	2020	0.80%	41	yes
He J et al. [30]	2018	10.00%	19	yes
Blair AB et al. [31]	2021	9.06%	30	yes
Perri G et al. [32]	2021	3.10%	9	Yes ^1^
Peng JS et al. [33]	2019	5.60%	4	no
Barrak D et al. [34]	2022	3.30%	177	yes
Mellon EA et al. [35]	2017	7.00%	6	yes
Truty MJ et al. [36]	2021	10.3%	20	yes
Hashemi-Sadraei N et al. [37]	2018	11.32%	6	no
Tamburrino D et al. [38]	2024	3.80%	15	yes
Abaydulla E et al. [39]	2025	12.76 ^2^	6	no
Jeon HJ et al. [40]	2022	12.5%	8	yes
Rashid OM et al. [41]	2016	14.5%	10	NR

## Data Availability

The original contributions presented in this study are included in the article. Further inquiries can be directed to the corresponding author.

## Abbreviations

The following abbreviations are used in this manuscript:

AJCC	American Joint Committee on Cancer
BR—PDAC	borderline resectable pancreatic cancer
CAP	College of American Pathologists
CA19-9	carbohydrate antigen 19-9
CEA	carcino-embryonic antigen
ChT	chemotherapy
CK	creatine kinase
CRT	chemoradiation
CT	computed tomography
CTCs	circulating tumor cells
ctDNA	circulating tumor DNA
EUS	endoscopic ultrasonography
fPR	favorable pathological response
fPRsp	favorable pathological response
gBRCAm	germline BRCA1 and/or BRCA2 mutation
ICI	immune checkpoint inhibitor
irRTV	immune-related residual tumor volume
LA—PDAC	locally advanced pancreatic cancer
LMR	lymphocyte-to-monocyte ratio
MMP-7	matrix metalloproteinase 7
MPD	main pancreatic duct
NAT	neoadjuvat treatment
NCCN	National Comprehensive Cancer Network
nCR	near complete response
NLR	neutrophil-to-lymphocyte ratio
Nsp	no response
NR	not reported
OS	overall survival
PatRsp	pathological response
PAX-G	scheme of chemotherapy: cisplatin, abraxane, capecytabine, gemcytabine
pCR	complete pathological response
PD-1	programmed death receptor 1
PD-L1	programmed death ligand 1
PDAC	pancreatic adenocarcinoma cancer
PFS	pregression-free survival
PLR	platelet-to-lymphocyte ratio
pMR	pathologic major response group
PR-PDAC	potentially resectable pancreatic ductal adenocarcinoma
PRsp	partial response
RAMPS	radical antegrade modular pancreatosplenectomy
RECIST	response evaluation criteria in solid tumors
RT	radiation
SBRT	stereotactic body radiation therapy
SIRI	systemic inflammatory response index
TNT	total neoadjuvant therapy
TRG	tumor regression grading system
ufPRsp	unfavorable pathological response
